# Investigating the impact on mental wellbeing of an increase in pensions: A longitudinal analysis by area-level deprivation in England, 1998–2002

**DOI:** 10.1016/j.socscimed.2022.115316

**Published:** 2022-08-30

**Authors:** Viviana Albani, Heather Brown, Esperanza Vera-Toscano, Andrew Kingston, Terje Andreas Eikemo, Clare Bambra

**Affiliations:** a Population Health Sciences Institute, Faculty of Medical Sciences, Newcastle University, Newcastle Upon Tyne, UK; b Division of Health Research, Lancaster University, Lancaster, UK; c Melbourne Institute of Applied Economic and Social Research, The University of Melbourne, Australia; d Department of Sociology and Political Science, Centre for Global Health Inequalities Research (CHAIN), Norwegian University of Science and Technology (NTNU), Dragvoll, Trondheim, Norway

**Keywords:** Social policy, Health inequalities, Deprivation, Social determinants, Mental health

## Abstract

In 1997 approximately two million people aged 60 years or over were living poverty in the UK. In 1999 the UK Government raised real pension incomes of low-income pensioners by around a third through the introduction of the Minimum Income Guarantee (MIG). This study explores the implications of this change for pensioners’ mental wellbeing with a focus on differences by area level deprivation in England. We explore mental wellbeing outcomes of 205 men (750 person-year observations) and 367 women (1,336 person-year observations) of state pension age from scores on the General Health Questionnaire from the British Household Panel Survey using a panel difference-in-difference estimation procedure. We compare the mental wellbeing of pensioners receiving MIG to that of low-income pensioners not claiming MIG, from 1998 to 2002. To investigate differences by area deprivation we use quintiles of the of the distributions of the 2000 and 2019 local-authority-level English Index of Multiple Deprivation. Models controlled for age, marital status and year. Between 1998 and 2002, 136 (38%) of low-income women and 57 (28%) of low-income men in the sample were claiming MIG at any one time. Income increased by 31% for men and 22% for women. There was no change in mental wellbeing for women but we found an improvement for men overall and for men living in the most deprived areas, in the latter case with a decrease of the GHQ-12 score of 2.43 points (95% CI: −5.49, 0.02). This estimate was similar across all measures of deprivation, and across both years of IMD. This study provides tentative evidence that the increase in pension income in England for low-income pensioners contributed to a reduction of inequalities in mental wellbeing for men. This needs to be considered in terms of future state pension policies.

## 1 Introduction

Rapid rates of population ageing across countries represent both challenges and opportunities for governments concerned about the health and healthy ageing of increasingly older populations (Greer et al., 2021). In addition to expenditure on health care and social care, welfare entitlements are another avenue through which governments can contribute to the health of their older citizens. Programmes or systems designed to alleviate poverty in retirement, such as non-contributory pension schemes, can improve the quality of life of low-income pensioners by increasing their household incomes and, through this mechanism, access to necessities, ability to cope better in emergencies and reducing financial worries (Green et al., 2017a; Moffatt and Scambler, 2008). This is important as less anxiety over finances, and better overall health have been correlated with lower levels of depression and psychosocial distress and higher mental wellbeing (Kim et al., 2016; Mendes De Leon et al., 1994).

In accordance with this, several studies across high and middle income countries have found positive effects of welfare reforms which increase pension income on the mental health of pensioners (for a systematic review see Simpson et al., 2021). These effects, however, are not uniformly recorded across social groups. In a study of an *ad-hoc* expansion in pension benefits for a US cohort of low-education pensioners, Golberstein’s (2015) instrumental-variables estimates showed a positive effect of an additional US$1,000 of Social Security income on depression scores and probability of experiencing depression in women, but no effects of the increase in incomes in men. In a similar context to our study, García and Otero (2017) applied a difference-in-difference estimator with instrumental variables and found positive effects for men of the introduction of a non-contributory means-tested pension for low-income individuals facing retirement in Chile, but no effects of the increase in expected pension wealth on women’s mental health outcomes. Differences have also been observed by education level of the household reference person, and by baseline level of cognitive health (Ayyagari and Frisvold, 2016). These and other potential heterogeneities in the effects of welfare reforms on mental health have implications for mitigating or intensifying health inequalities in old age.

In England, men and women over the age of 65 residing in the most deprived neighbourhoods can expect to live on average five years less than their peers in the least deprived areas of the country (Office for National Statistics, 2021). Simultaneously, while pensioners’ poverty is less localised than poverty in other age groups, it tends to concentrate spatially in more deprived areas (Fransham, 2018). This geographical heterogeneity among the elderly arises from individual and spatial inequalities in the distribution of the social determinants of health (e.g., income, employment, housing, access to health care, etc.) (Bambra, 2016; Pearce, 2013; Vera-Toscano et al., 2020) over the life course. To the extent that welfare reforms - such as changes to pension income - affect these underlying factors, they can be expected to impact area-level health inequalities in old age. The relationship between public transfers and inequalities in health in the elderly remains under-explored within the literature, particularly in terms of inequalities in mental and psychological wellbeing by area deprivation. Differences in the effect on individuals’ wellbeing of a change in income by area deprivation may be expected from a materialist perspective. More specifically, an increase in pensions may have a greater effect on the wellbeing for pensioners with the lowest incomes because this group would have had more difficulties in accessing crucial everyday necessities that now become more affordable (Cummins, 2000; Sareen et al., 2011). Alternatively, differences by area deprivation may also be expected from a psychosocial perspective. That is, seeing one’s income increase compared to others in the neighbourhood (Ferrer-i-Carbonell, 2005) – as well as feeling more financially secure. This effect potentially will be stronger when it is a low-income neighbourhood (Neman, 2020).

This paper aims to examine the effect of a Minimum Income Guarantee (MIG) – introduced in 1999 - on the mental and psychological health of low-income pensioners, paying particular attention to how the size of the estimated effect varies by area of deprivation. Previous studies in the UK have focused on the impact of fiscal austerity measures after the Great Recession (post-2007/8) on increasing old age mortality (Loopstra et al., 2016; Green et al., 2017b). However, the authors did not report on inequalities by area level deprivation. Interestingly, Akhter et al. (2018) did not find any significant change after introducing these austerity measures in the mental health gap between the least and the most deprived areas in a locality in the North East of England. The authors suggested that the protection afforded to pensioners’ income from the UK “Triple Lock” system - designed to protect pensioner income growth - could have accounted for the observed stability in the gap in mental health. To the best of our knowledge, this paper is the first study that contributes empirical evidence of the effect of the expansion of welfare policies on inequalities by area level deprivation among the elderly across England.

To meet this aim, we use the case of the 1999 UK pension reform known as the Minimum Income Guarantee (MIG), which increased the pension income of low-income retired persons by around a third, lifting approximately two million pensioners out of poverty. Our empirical analysis offers two critical insights. First, we empirically evaluate whether welfare policies impact on the mental wellbeing of pensioners living in the most deprived areas. Secondly, our contribution also engages with the call to ‘scale up’ research into geographical inequalities in health by rigorously studying how macro-level factors such as welfare and economic policies influence geographical patterns of health and wellbeing (Bambra et al., 2019).

### 1.1 The Minimum Income Guarantee

In 1997 the UK’s Labour Government set out plans to meet its aspiration of decent and secure income in retirement. Among its targets, the Government was keen to combat pensioner poverty. By its own calculations, at least two million people aged over 60 were experiencing poverty, with incomes below income support (IS) rates (a means-tested non-contributory cash benefit designed to provide individuals with a minimum level of income) (UK Parliament Select Committee on Work and Pensions, 2002). In 1999, the Government introduced the Minimum Income Guarantee (MIG) for pensioners (Bozio et al., 2010). This was simply equal to the existing IS personal allowance plus the pensioner premium, but the rebranding was accompanied by an increase in the pensioner premium of three times the normal increase, which made it a distinct policy change. MIG was targeted at low-income households with at least one individual aged 60 years or over (UK Parliament Select Committee on Work and Pensions, 2002), and was designed to increase weekly income to a guaranteed level set to increase each year broadly in line with earnings. All pensioners claiming income support before the introduction of the MIG were automatically eligible for the increase in pension income associated with the reform. However, as with the IS, the MIG had to be claimed, and take-up was not 100%, meaning that pensioners who did not claim may still have had income below the minimum level that was being guaranteed by the state. At its start, in April 1999, weekly MIG rates were set at £75.00 for 60–74 year olds (£116.60 for couples), £77.30 for 75–79 year olds (£119.85 for couples) and £82.25 for those 80 years of age or over (£125.30 for couples) (UK Parliament Select Committee on Work and Pensions, 2002). As with the overarching IS scheme, any income above the government-set threshold resulted in a £1 for £1 reduction in MIG benefits (100% effective marginal withdrawal rate), and therefore a disincentive to claim MIG at incomes above the threshold (Bozio et al., 2010), but also potentially a disincentive to save for retirement (Brewer and Emmerson, 2003).

The IS pensioner premium increase that resulted from the introduction of the MIG was substantial. From 1997 to 2002, the increase in the maximum amount of pension for a prototypical single-pensioner household under the age of 75 years grew in real terms by 31%, compared to an increase of 6% in the period from 1992 to 1997, representing the third highest change in benefits in real terms after that for lone parents (Brewer et al., 2002). Over this period, a particularly large increase in the MIG happened in April 2001 when all the different MIG rates by age bands were matched to the highest threshold, which in turn was increased by that year’s real increase in the basic state pension and by average earnings growth (Bozio et al., 2010). Also, in April of 2001, the capital limits to be eligible for the MIG were increased, doubling the lower limit from £3,000 to £6,000 and increasing the upper limit from £8,000 to £12,000 (UK Parliament Select Committee on Work and Pensions, 2002).

## 2 Methods

### 2.1 Data

Data for analyses come from the British Household Panel Survey (BHPS) covering the period 1998–2002 (University of Essex et al., 2017). The BHPS is a nationally representative longitudinal panel survey based on a two-stage stratified clustered random sample of households from the four UK countries from 1991 to 2008. Most responses in the BHPS were collected face-to-face by trained interviewers. Relevantly for this study, the BHPS did not survey people living in institutions (University of Essex, 2017). Each survey year in the BHPS represented a survey wave, with most interviews carried out between September and November of a given year. This timeframe allows us to explore the impact of changes in income from one year to the next as increases in MIG occur in April of each year. Given the start year of MIG in 1999, we restrict the sample to start on the 1998 wave to capture the pre-reform effects, we use data only until 2002 as in 2003 the MIG was replaced with a different pension benefit scheme (Pension Credit) with wider eligibility criteria to those applied for MIG receipt (Bozio et al., 2010). The study sample comes from the 2,166 individuals taking part in the English sample of the BHPS during the above years. Each individual respondent might have participated up to 5 times between 1998 and 2002. Information on the local authority (LA, see below) where BHPS respondents lived came from a special license version data that is part of the restricted access information gathered by the survey (University of Essex, 2020).

### 2.2 Outcome measure

The outcome variable is mental wellbeing, measured as the aggregate score on the twelve-part General Health Questionnaire (GHQ-12) (Goldberg et al., 1997) collected annually. The GHQ-12 scale has been shown to be a reasonably reliable instrument for assessing mental health symptoms in the general population (Elovanio et al., 2020; Goldberg et al., 1997). The scale covers twelve items which can be found in the online supplementary material. The GQH-12 score resulting from the answers to the 12 questions ranges from 0 (highest possible wellbeing) to 36 (lowest possible wellbeing). More precisely in the context of this study, the GHQ-12 variable is a good instrument to explore the relationship between income and psychological/mental wellbeing if we expect changes in income to affect individuals through psychosocial mechanisms of stress and anxiety (Jones and Wildman, 2008).

### 2.3 Index of Multiple Deprivation

Heterogeneity by area level deprivation in policy effects was explored using the Local Authority district (LA) level 2000 Index of Multiple Deprivation (IMD) for England (Firth and Payne, 1999a, b). LAs is the local administrative body in English government. They comprise various different geographies, including Non-metropolitan Districts, Unitary Authorities, London Boroughs and Metropolitan Districts (Rabe, 2011). The LA 2000 IMD is constructed by aggregating data from 8,414 wards collected from 1998 to 1999, i.e., at the time of the MIG reform, on domains of deprivation in terms of income; employment; health and disability; education skills and training; housing; and geographical access to services. Because LAs are large geographic areas that can contain both pockets of high and very low deprivation, we follow Firth and Payne (1999b) in using three different measures of the 2000 IMD at the LA level:

- **LA average deprivation**. This is the overall measure of deprivation across a LA and it is calculated as the population weighted average of the IMD scores for the wards in a LA. This approach may mask high deprivation areas within LAs
- **LA extent of deprivation** portrays how widespread high levels of deprivation are within a LA. It is constructed as the proportion of a LA’s population living in the wards which rank within the most deprived 10% of wards in England. The higher the proportion, the more deprived the LA is on this measure. A caveat of the extent of deprivation is that LAs that do not include one of the ten percent most deprived LA in the country all receive the same score, irrespective of their deprivation level.
- **LA concentration/intensity of deprivation** is calculated as the population-weighted average of scores of a LA’s most deprived wards that together represent the 10% of the district’s population. The local concentration of deprivation therefore compares how much more deprived are the most deprived areas across LAs (i.e., the acuteness of deprivation in the most deprived areas of an LA) and is therefore an important way of identifying LA with ‘hot spots’ of deprivation.

For the analyses of LA average and concentration/intensity deprivation, LAs were split into quintiles of the distribution of the deprivation score. For the analysis of LA extent of deprivation, LAs were split into the 20% most deprived and the rest of the country (80%) since, given the nature of the indicator, more than 50% of LAs had similar deprivation score.

Heterogeneity by area level deprivation in policy effects was explored using the local authority 2000 and 2019 Index of Multiple Deprivation (IMD) for England (Firth and Payne, 1999a, b; Penney, 2019). The 2000 IMD was constructed using data from 1998 to 1999 from 8,414 wards across its different constituent indicators (i.e., at the time of the MIG reform) and included domains of deprivation in terms of income; employment; health and disability; education skills and training; housing; and geographical access to services. The 2019 IMD is based on data ranging from 2015 to 2019 (some indicators use 2011 Census) for 32,482 Lower-layer Super-Output Areas (LSOAs) and includes domains on income deprivation, employment deprivation, health deprivation and disability, barriers to housing and services, crime, and living environment deprivation. Although conceptually the domains remain similar across the two versions of the IMD, the 2019 IMD represents an extensively revised version of the 2000 IMD and the most up to date definition of local area deprivation for England. In other words, the 2000 IMD explores the effect on subjective wellbeing inequalities at the time of the reform, whereas the 2019 IMD is used as a robustness check on the findings.

LA summary measures of deprivation and their corresponding quintiles at the 2000 and 2019 LA district level were then matched to 2013 LA codes used in the BHPS geographical identifier variable (Rabe, 2011).

It is worth noting, that deprivation is an area level rather than an individual measure. There is evidence (Norman and Boyle 2014), that inequalities decrease as people age; however, since we control for area level deprivation changing deprivation profiles as people age should not affect our results.

### 2.4 Participants

We use data from all respondents between 1998 and 2002 who were of pensionable age in 1999. For women that is 60 years old and for men it is 65 years old (n = 2416 observations for men and n = 5211 observations for women). Next, we restrict our sample to those people who are low income. Following official government documents and previous literature, we defined low-income individuals as those living in a household with an income below 60% of the median value of household incomes in the full sample in any given year (UK Parliament Select Committee on Work and Pensions, 2002; Vera-Toscano et al., 2020). Household income was converted to real terms using the UK 2015 consumer price index (Office for National Statistics), and equivalised by dividing total household income by the number of people in the household. This is in contrast to the method used by the UK Department for Work and Pensions in its estimates of the Households Below Average Income statistics, which use two child-less persons as reference household, and gives a weight of 0.67 to a single adult. Our approach instead uses as reference a one person household without children, as in 1998 the BHPS sample 25% of men aged over 65 years were living in single pensioner households, but 47% of women aged 60 years or over were living alone. This reduces our sample to n = 966 observations for men and n = 1675 women. We conduct a complete case analysis, so we require full information on GHQ-12, local authority area of residence, and information on if the person receives MIG. This gives us our final estimation sample of n = 750 observations for men and n = 1336 observations for women.

### 2.5 Estimation strategy

We examine the impact of the increase in pension income for low-income pensioners using a difference-in-difference (DiD) approach. This approach has been widely applied to study the impact of policy reforms to estimate causal effects in observational studies, i.e., when it is not possible to randomly assign individuals to a new policy/policy change (treatment) (Wickham et al., 2020; Wing et al., 2018). The method compares the change in the outcome of interest over time in the group exposed to the policy change (e.g. receiving increased benefits) to the change in outcome over time in a comparable group not affected by the reform. The assumption is that this approach identifies the change in mental wellbeing that is due to the reform as both groups are affected in the same way by other time-varying influences on mental wellbeing, in effect treating the comparison group as an approximation to the (counterfactual) change in health of the affected group in a scenario without the reform (for a general exposition, see e.g., Angrist and Pischke, 2009; Stuart et al., 2014). Here we compare the change in mental wellbeing after the introduction of the MIG between MIG recipients and non-recipients. Specifically, individuals exposed to the policy change (“treated”) were those receiving income support in 1998 based on responses to the item on social benefits income “receives income support” collected in the BHPS from 1991 onwards. The control group were low-income pensioners that did not claim income support in 1998. If individuals stopped receiving MIG in one of the post-treatment years, they were assigned to the control group for that year, and if respondents started receiving MIG in subsequent post-treatment years, they were classed as treated in that wave (Wickham et al., 2020). By taking this flexible approach to the control and treatment group over the study period our results will be a conservative estimate of the impact of MIG on mental health. Following official government documents and previous literature, we defined low-income individuals as those living in a household with income below 60% of the median value of household incomes in the full sample in any given year (Department for Work and Pensions; Vera-Toscano et al., 2020). Household income was converted to real terms using the UK 2015 consumer price index (Office for National Statistics) and equivalised by dividing total household income by the number of people in the household.

The estimation strategy for the DiD design is based on equation (1) for wellbeing outcome *Y*_*it*_ for individual *i* at time *t* (see e.g. Miller, 2012). We employ a panel regression with fixed effects estimated by OLS to remove the bias from omitted variable bias on individual characteristics, but not the treatment variable. The fixed effects estimator, also called the within estimator, is assumed to control for time-constant unobserved individual characteristics that would influence MIG take up over time, attenuating the possibility that the observed effect from Equation (1) of the policy on mental wellbeing is biased by the characteristics of pensioners that received income support compared to those that were eligible but did not receive it (see Toynbee and Walker, 2011 p.162; Wooldridge, 2010 p.289, 315).

(1) $$Y_{it}=\eta_{i}+\tau P_{it}^{*}D_{it}+\sum_{t=1999}^{2002}\delta_{t}T_{t}+\beta^{\prime }X_{igt}+\varepsilon_{igt}$$

*η*_*i*_ are individual specific intercepts, *D*_*it*_ is an indicator variable identifying individuals affected by the policy change, in this case, people receiving income support; *P*_*it*_ is an indicator variable for the pre-policy reform (1998) and post-policy reform periods (1999–2002) for individual *i*; *P*_it_*D_*it*_ represents individuals receiving MIG in the post-treatment period, and the coefficient *τ* corresponds to the difference-in-difference estimate of the effect of the policy reform. Controlling for individual fixed effects contributes to reducing feedback effects (Wooldridge, 2010 p. 289), and additional tests excluding the sample of individuals that switch groups affected power but did not indicate issues with bias (i.e., large differences in estimates). *T*_*t*_ are year indicators {*t* = 1999, …2002} capturing common trends over time across pensioner groups (time fixed effects) and *X*_*igt*_ are individual time-varying characteristics of age in years and pensioner household type. Age categories were defined as 60–64, 65–69, 70–74,75-79,80 or more years to reflect non-linear effects of age on well-being (Wickham et al., 2020). Pensioners were classed as living alone or in a couple, as being a lone pensioner can influence both wellbeing and household income level (Kim et al., 2016). Finally, *ε*_*igt*_ is a stochastic error term varying by individual, treatment group and time. Standard errors were estimated using 1,000 bootstrap replications stratified by region (“North and Midlands”, “South”) with errors clustered at the level of the individual respondent to account for correlations over time in individual-level observations and cross-sectional heteroskedasticity (Autor, 2003).

To explore the inequality effects of the policy change, model (1) with panel fixed effects was estimated separately for observations across quintiles of LA average IMD, extent of IMD and concentration IMD. We follow previous literature and stratify our analyses by sex to account for potential differences between men and women in rates of depression and other mental disorders (e.g., Golberstein, 2015) and because we expected men and women to have different pension histories and therefore potentially different effects from the introduction of MIG.

A critical assumption for the success of the DiD approach is that the groups being compared share a similar trend over time in the outcome of interest before the policy change, referred to as the parallel trends assumption (Wing et al., 2018). If this is the case, then the observed changes in the treated group after the introduction of a policy can be attributed to the effect of the policy as other time-sensitive factors that could influence the outcome would have been factored out using the post-intervention change in the control group (Wing et al., 2018).

With data available for more than one period before the introduction of a policy, it is possible to test if the treatment and control groups had differing trends before the start of the reform, providing some reassurance of the suitability of the DiD (Wickham et al., 2020). We test this using the DiD framework detailed below but interacting the treatment group with 1991–1998 survey waves. We found no significant differences between the two groups, for men and women separately, across the 1991 and 1998 waves (Table A1 in Appendix).

## 3 Results

The data set for analyses included 205 (35%) men with 750 person-year observations, and 367 (65%) women with 1,336 person-year observations. Fifty seven percent (57%) of individuals were observed in all survey waves and 75% of individuals had three to four observations in the study period after the introduction of MIG. Between 1998 and 2002, 136 (38%) of women and 57 (28%) of men were claiming income support at any one time. Across the entire sample period, there were more cases of oldest old women (80 years or more) receiving income support compared to those not receiving it (36% vs 23%, Chi^2^[4] = 29.91, *p-value*<0.001), a trend that was repeated for men (36% vs 24%, Chi^2^[3] = 13.13, *p-value* = 0.004). Also, across the full sample, in most cases, women over state pension age were living alone (61%), while in most cases men were living with a partner (72%). However, the proportion of women living alone was much higher in cases claiming income support (94%), with about two-thirds of cases of men claiming income support living alone (60%). For cases claiming income support, 42% of observations were from the most deprived fifth of LADs compared to 29% for those not claiming income support (Chi^2^[4] = 65.84, *p-value* < 0.001).

Between 1998 and 2002, the average real income of men pensioners receiving income support increased by £196 per month with the introduction of MIG (Table 1). The average increase for women was slightly lower at around £160 per month. Pensioners not receiving MIG also saw their real average monthly income increase during this period, at around £115 per month of additional pension income because of concurrent increases to the government’s state pension (UK Parliament Select Committee on Work and Pensions, 2002). On average, there was no change in men’s and women’s mental wellbeing scores (results were not statistically significantly different from zero). Interestingly, pensioners receiving MIG, both women and men, reported lower mental wellbeing than pensioners not claiming MIG. Looking more closely at the data (results not shown), the worse mental wellbeing at baseline in MIG recipients was concentrated in those with no educational qualifications (both men and women) and for men pensioners not living in partnership. Table A2 and Table A3 in the Appendix provide mean values of income and mental wellbeing by quintile of IMD.

Results of the DiD OLS estimation on the effect of the MIG on mental wellbeing for men and women are presented in Table 2. Table 3 and Table 4 show the estimates stratified by 2000 IMD quintiles, while Table 5 and Table 6 present results for 2019 IMD quintiles.

Overall, mental wellbeing in men showed signs of improving after the introduction of MIG (a GHQ-12 lower score by 1.84 points, 95% CI: −3.86, 0.23), but no change in mental wellbeing after the introduction of the MIG was observed for women. Looking at area deprivation according to the 2000 IMD score, we find heterogeneity in the effects of the reform for men pensioners. In this case, the average GHQ-12 score for men pensioners living in the most deprived areas was −2.43 (95% CI: −5.49, 0.02) after the reform, suggesting improvement in average psychological/mental wellbeing for this group. The effect was similar when considering the extent of LAD deprivation (Table 3). For the LAD average deprivation and LAD concentration of deprivation measures, coefficients for the third and fourth most deprived quintiles were also negative, suggesting a positive effect of the introduction of MIG on psychological/mental wellbeing also for these areas and, therefore, on regional health inequalities. Still, for most of these estimates, confidence intervals are very large, spanning both high negative and positive change values, precluding any concrete interpretation of the direction of effects. Also, for the LAD average deprivation and LAD concentration of deprivation measures, the introduction of the MIG instead amplified poor psychological/mental wellbeing for men in the second least deprived LADs, with, on average, an increase in the GHQ-12 score ranging from 3.5 (95% CI: 1.56, 5.38) to 4.4 (95% CI: 0.4, 10.06) points. Results were broadly similar when examining inequalities by 2019 IMD LAD indicators, with improvements in psychological/mental wellbeing in the 4th and most deprived quintiles on the measures of LAD average and LAD extent deprivation; and increases in inequality in the least deprived quintiles (Table 5).

In the case of women, we did not find a clear pattern of changes in psychological/mental wellbeing by 2000 IMD deprivation scores (Table 4), with inequalities reducing by LAD concentration in the second least deprived quintile but increasing in the third quintile. There were no other significant differences in psychological/mental wellbeing for this group, with large standard errors possibly accounting for the null findings (Table 4). For 2019 IMD scores, we found no differences in psychological/mental wellbeing across the quintiles of deprivation (Table 6).

## 4 Discussion

This study explored inequalities by area level deprivation in the impact on pensioner psychological/mental wellbeing of a large increase in pension income affecting low-income households in England. The effect of public transfers on the health outcomes of recipients, particularly mental health outcomes, has received much attention in the literature, but less so for older people, and even less on the impact of health inequalities in old age (Simpson et al., 2021). We found no effect on the mental wellbeing of the increase in household income resulting from the reform for the overall sample of women and a weak effect in men. By deprivation at the area level, we found both an improvement in the mental wellbeing of men living in the most deprived areas using two temporally different measures of area deprivation, and a deterioration of mental wellbeing of men living in the least deprived areas of England, but no effects for other less deprived groups of men, and no effects for women.

Our findings contrast with the result from a USA study (Golberstein, 2015), which noted an improvement in mental health in women from a US$1,000 increase in annual pension income. This was explained as a result of the support for independent living that the extra income afforded to women. However, in Golberstein’s study, women experienced an 86% increase in income compared to an increase of 11% for men. This was much larger than in our study where women received a smaller increase of 22% compared to 31% for men. Additionally, the different health care systems and eligibility for services may also contribute to the conflicting findings between the studies. Our results are more in line with studies from South Korea, where similar shifts in income - that were not considered sufficient to affect pensioners’ financial security also had null findings for wellbeing (Lee and Wolf, 2014; Pak, 2020). Also at play in our results could be gendered differences in the importance of income, where studies have suggested that income is more important to men’s wellbeing because of cultures of male bread-winning (Ashwin et al., 2021).

Our results suggest that men living in the most deprived areas of England experienced an improvement in their mental wellbeing after the introduction of MIG. This result was consistent across the 2000 and the 2019 IMD scores, and all three measures of LAD deprivation used. This may reflect that a boost for men with the lowest incomes improves access to crucial material benefits previously out of reach (Cummins, 2000; Sareen et al., 2011). The psychosocial effect of seeing one’s income increase compared to others locally may also be important. A study on Chinese pensioners found that those who received a non-contributory pension perceived themselves to be richer than others in their local area, and that this explained lower depression scores observed for this group (Cheng et al., 2018). Separate evidence indicates that this effect can be more substantial in a low-income neighbourhood (Neman, 2020). Our study has provided an example of how macro-level policies can impact geographical health inequalities (Bambra et al., 2019).

### Strengths and limitations

A strength of this study is its use of individual-level data over time to look at geographical health inequalities. Our approach allowed us to control for time-constant individual differences that may have otherwise biased our results. Our approach also allowed us to address changes over time in the mental health of pensioners not associated with the introduction of MIG, providing greater reassurance on a causal interpretation of our findings. In addition, previous work on the relationship between income and health in older populations using individual-level data has left the topic of geographical inequalities largely unexplored. Other strands of research looking at geographical health inequalities have mostly used area-based aggregate measures, which are potentially subject to bias introduced by averaging over population groups. In terms of limitations, we remark on the small sample sizes in the analyses by area deprivation. We chose to split the sample by quintiles of deprivation to expose deprivation gradients. This may have contributed to the large confidence intervals observed, reducing the power to detect real improvements in mental wellbeing, for example, as seen for the mental health estimates in men living in the third and fourth quintiles of deprivation. The scale at which we measured deprivation – local authority – may also have limited our findings. There are some limitations with using a self-reported measure of mental wellbeing to study the relationship between income and mental health. Difficulties arise in understanding the effect of income on health if there is systematic error in reporting of mental health issues precisely according to income level. This can happen, for example, when people with different income levels have different understandings of health, different access and use of health care services and thus different levels of accurate information about their health status, as well as different expectations of what their health should be (e.g., Jones and Wildman, 2008). We did not have access in our data to more objective measures of mental wellbeing (e.g., medical records of prescriptions for anti-depressant drugs). However, we use a widely validated psychometric scale of mental well-being in general populations that does not directly ask questions on health or health conditions, but on everyday lived experiences (e.g., loss of sleep) with reference to oneself, which may be less susceptible to some of these biases. As with other studies using longitudinal samples with older participants, loss to follow-up is a realistic expectation as older individuals die or become too sick to take part in surveys. Given the short time span covered in our study, however, around 75% of our sample had complete or almost complete follow-up observations, with the largest drop-out rate in those older than 80 years and, in particular, women (data not shown). Extant research on wellbeing and age suggests that with increasing age wellbeing increases, irrespective of income, and income has less effect on wellbeing at older ages (Hsieh, 2011; Sareen et al., 2011; Schwandt, 2016). Further, our results may not be generalisable to those over 80.

## 5 Conclusion

This study contributes initial evidence that an increase in pension income for low-income pensioners can help reduce health inequalities by deprivation for men. Results also highlight the need to understand better the drivers and protective factors of mental wellbeing in retired women. Findings from this study have implications for public policy in the context of aging populations. The ongoing debates about how to fund the cost of the economic and health impacts of the 2020–2021 COVID-19 pandemic have brought forward revisions on governments’ taxation and welfare expenditures, including the cost of pension schemes. In the UK, debate has intensified around abandoning the existing triple-lock system, which was designed to protect pensioners’ income by increasing state pensions by the largest of the inflation rate, the earnings growth rate or 2.5%. During a period of escalation of the discussions around changes to entitlements for the elderly that will most likely see a reduction in benefits (see e.g., Emmerson, 2020), it is important to consider implications to inequality in the wellbeing of pensioners.

## Supplementary Material

**Appendix A. Supplementary data**

Supplementary data to this article can be found online at https://doi.org/10.1016/j.socscimed.2022.115316.

## Figures and Tables

**Table 1:** Mean monthly income and psychological/mental wellbeing scores (GHQ-12) by pensioner group, before and after the introduction of MIG.

		Men				Women		
		Low-income household with income support (MIG)		Low-income household with no income support		Low-income household with income support (MIG)		Low-income household with no income support
**Mean real (2015 GBP) monthly equivalised household income**								
Before		575.8 (187) [501.3]		486.9 (84.1) [505.4]		690.7 (333.2) [560.3]		488.3 (80.5) [495]
After		771.8 (280.1) [736.9]		599.7 (197.6) [565.9]		848.5 (281.5) [823.7]		607 (218.6) [565]
Difference		196.0***		112.8***		157.7***		118.7***
Change (%)		34.0		23.2		22.8		24.3
**Mean GHG-12 score, range 0 (best health) - 36 (poor health)**								
Before		12.55 (7.09) [6]		10.58 (4.87) [4]		13.1 (5.2) [3]		11.59 (4.04) [4]
After		11.7 (4.88) [0]		10.85 (5.03) [4]		13.36 (5.52) [3]		12.03 (5.07) [1]
Difference		−0.85		0.27		0.30		0.40
Change (%)		−6.8		2.6		2.3		3.5

Standard deviations in parenthesis. Median income and minimum value of the GHQ-12 score in square brackets. Test of difference in income from a OLS regression of the log of real income on receipt of income support and pre- and post-policy period. Test of difference in GHQ-12 scores from a tobit regression with “zero” left-censoring value on receipt of income support and pre- and post-policy period. All standard errors clustered at the individual respondent level. Difference between before and after period significant at: *** 1% level, ** 5% level, and * 10% level.

**Table 2:** Estimates of psychological/mental wellbeing (GHQ-12 score) by pensioner group after the introduction of the Minimum Income Guarantee.

	Men				Women		
	Coef.	p-value	95% CI		Coef.	p- value	95% CI
MIG	0.30	0.801	(-1.92, 2.71)		−0.08	0.919	(-1.78, 1.21)
MIG x Post	−1.84	0.075	(-3.86, 0.23)		0.27	0.630	(-0.84, 1.44)
*σ_u_*	4.68				4.46		
*σ_e_*	3.04				3.24		
Total	750				1336		
Clusters	207				372		

95% CI in parentheses. *σ_u_* is the between person standard error. *σ_e_* is the model-wide error. Regression results from a model controlling for year dummies, age categories and living in a couple. Estimates from a panel fixed effects OLS regression. Standard errors from 1,000 bootstrap replications with cluster standard errors at the individual level and stratified by geographic area.

**Table 3:** Estimates of psychological/mental wellbeing (GHQ-12 score) by pensioner group after the introduction of the Minimum Income Guarantee. By 2000 IMD quintiles. Men.

		Average^a^						Extent						Concentration				
		Coef.		p-value		95% CI		Coef.		p-value		95% CI		Coef.		p-value		95% CI
**Least deprived**																		
MIG		−0.99		0.623		(-4.54, 2.42)		−0.28		0.84		(-2.86, 2.77)		−1.42		0.269		(-3.44, 0.19)
MIG x Post		−0.5		0.874		(-6.61, 2.3)		−1.39		0.345		(-4.65, 1.08)		−1.88		0.495		(-7.36, 0.08)
*σ_u_*		4.08						4.71						5.12				
*σ_e_*		2.99						3.07						2.93				
Total		101						505						107				
Clusters		29						138						31				
**2nd quintile**																		
MIG		−5.27		0.022		(-6.97, 1.63)								−4.89		0.039		(-9.79, −0.82)
MIG x Post		3.5		0.005		(1.56, 5.84)								4.35		0.075		(0.4, 10.06)
*σ_u_*		4.85												4.39				
*σ_e_*		2.71												2.76				
Total		124												136				
Clusters		35												35				
**3rd quintile**																		
MIG		0.55		0.825		(-3.45, 6.37)								3.59		0.164		(-0.86, 8.93)
MIG x Post		−2.5		0.379		(-8.65, 2.68)								−5.74		0.05		(-11.4, −0.13)
*σ_u_*		5.5												6.22				
*σ_e_*		3.54												4.07				
Total		162												114				
Clusters		41												32				
**4th quintile**																		
MIG		1.72		0.548		(-2.6, 7.1)								−0.49		0.888		(-7.0, 6.61)
MIG x Post		−2.68		0.356		(-10.1, 0.14)								−1.4		0.641		(-6.92, 4.25)
*σ_u_*		5.48												4.39				
*σ_e_*		2.59												2.62				
Total		132												178				
Clusters		38												48				
**Most deprived**																		
MIG		1.75		0.453		(-2.38, 6.72)		1.68		0.475		(-2.38, 7.31)		1.10		0.629		(-2.62, 6.07)
MIG x Post		−2.43		0.071		(-5.49, 0.02)		−2.75		0.077		(-6.47, −0.21)		−1.84		0.164		(-4.69, 0.43)
*σ_u_*		4.83						4.88						4.68				
*σ_e_*		2.93						2.95						2.88				
Total		231						245						215				
Clusters		65						68						62				

a Average, Extent and Concentration refer to the Average, the Extent of deprivation and Concentration of deprivation IMD aggregate indicators by local authority. 95% CI in parentheses. *σ_u_* is the between person standard error. *σ_e_* is the model-wide error. Regression results from a model controlling for year dummies, age categories and living in a couple. Estimates from a panel fixed effects OLS regression. Standard errors from 1,000 bootstrap replications with cluster standard errors at the individual level and stratified by geographic area.

**Table 4:** Estimates of psychological/mental wellbeing (GHQ-12 score) by pensioner group after the introduction of the Minimum Income Guarantee. By 2000 IMD quintiles. Women.

		Average^a^						Extent						Concentration				
		Coef.		p-value		95% CI		Coef.		p-value		95% CI		Coef.		p-value		95% CI
**Least deprived**																		
MIG		1.72		0.754		(-6.91, 11.49)		0.77		0.441		(-1.2, 2.74)		5.44		0.206		(-5.52, 12.8)
MIG x Post		−0.21		0.928		(-6.25, 3.35)		0.18		0.776		(-1.05, 1.47)		0.17		0.849		(-1.66, 1.92)
*σ_u_*		4.69						4.62						4.64				
*σ_e_*		2.7						3.21						2.42				
Total		147						870						151				
Clusters		42						238						42				
**2nd quintile**																		
MIG		1.35		0.417		(-1.96, 4.46)								0.78		0.769		(-3.22, 7.95)
MIG x Post		0.51		0.532		(-0.77, 2.64)								−2.01		0.079		(-4.35, 0.19)
*σ_u_*		4.32												5.05				
*σ_e_*		2.42												3.52				
Total		204												204				
Clusters		56												58				
**3rd quintile**																		
MIG		1.21		0.422		(-1.78, 4.14)								−2.09		0.088		(-4.45, 0.08)
MIG x Post		−1.03		0.238		(-2.85, 0.54)								3.28		0.015		(0.96, 6.31)
*σ_u_*		4.43												4.15				
*σ_e_*		3.61												2.98				
Total		334												227				
Clusters		93												63				
**4th quintile**																		
MIG		0.01		0.998		(-4.11, 2.51)								2.19		0.152		(-0.77, 5.18)
MIG x Post		2.36		0.150		(-0.3, 6.03)								−0.59		0.611		(-3.29, 1.31)
*σ_u_*		5.26												4.66				
*σ_e_*		3.67												3.34				
Total		207												302				
Clusters		59												86				
**Most deprived**																		
MIG		−1.53		0.220		(-1.85, 2.18)		−1.31		0.298		(-3.83, 0.96)		−1.34		0.268		(-3.76, 0.89)
MIG x Post		0.17		0.873				0.27		0.799		(-1.75, 2.45)		0.04		0.972		(-1.89, 2.17)
*σ_u_*		4.85						4.4						4.97				
*σ_e_*		3.08						3.23						3.29				
Total		444						466						452				
Clusters		125						130						124				

a Average, Extent and Concentration refer to the Average, the Extent of deprivation and Concentration of deprivation IMD aggregate indicators by local authority. 95% CI in parentheses. *σ_u_* is the between person standard error. *σ_e_* is the model-wide error. Regression results from a model controlling for year dummies, age categories and living in a couple. Estimates from a panel fixed effects OLS regression. Standard errors from 1,000 bootstrap replications with cluster standard errors at the individual level and stratified by geographic area.

**Table 5:** Estimates of psychological/mental wellbeing (GHQ-12 score) by pensioner group after the introduction of the Minimum Income Guarantee. By 2019 IMD quintiles. Men.

		Average^a^						Extent						Concentration				
		Coef.		p-value		95% CI		Coef.		p-value		95% CI		Coef.		p-value		95% CI
**Least deprived**																		
MIG		−1.57		0.416		(-5.55, 1.9)		−6.22		0		(-8.38, −3.22)		−6.08		0		(-7.96, −3.23)
MIG x Post		−0.24		0.94		(-6.67, 2.79)		4.2		0.025		(1.77, 8.09)		3.81		0.026		(1.65, 7.00)
*σ_u_*		4.07						5.79						5.56				
*σ_e_*		2.87						3.38						3.31				
Total		111						96						110				
Clusters		32						28						31				
**2nd quintile**																		
MIG		−2.45		0.261		(-6.5, 2.33)		−1.11		0.585		(-3.97, 4.45)		−0.68		0.729		(-3.63, 4.04)
MIG x Post		1.86		0.334		(-2.13, 5.83)		0.73		0.739		(-5.48, 3.92)		0.18		0.929		(-5.64, 3.32)
*σ_u_*		4.69						4.76						4.83				
*σ_e_*		3.67						2.4						3.25				
Total		113						152						138				
Clusters		29						39						36				
**3rd quintile**																		
MIG		0.59		0.852		(-4.8, 8.45)		0.38		0.933		(-8.15, 9.23)		5.29		0.254		(-1.16, 19.2)
MIG x Post		−2.97		0.357		(-9.77, 3.07)		−2.9		0.457		(-10.21, 3.7)		−6.03		0.198		(-19.4, 0.7)
*σ_u_*		5.23						4.8						5.07				
*σ_e_*		2.97						3.52						3.09				
Total		203						160						106				
Clusters		55						45						28				
**4th quintile**																		
MIG		4.4		<0.001		(2.56, 5.88)		3.2		0.051		(0.29, 6.64)		−1.15		0.810		(-9.99, 7.68)
MIG x Post		−5.44		<0.001		(-7.74, −2.67)		−6.09		0.019		(-11.3, −1.65)		−2.82		0.612		(-10.9, 10.3)
*σ_u_*		5.64						4.95						4.03				
*σ_e_*		1.94						2.42						2.5				
Total		67						91						140				
Clusters		19						24						39				
**Most deprived**																		
MIG		2.19		0.488		(-3.23, 8.42)		1.84		0.564		(-2.89, 9.25)		2.41		0.338		(-5.2, −0.27)
MIG x Post		−2.6		0.044		(-5.19, −0.18)		−2.41		0.104		(-5.95, 0.07)		−2.42		0.05		
*σ_u_*		4.77						4.73						4.8				
*σ_e_*		3.01						3.03						2.98				
Total		256						251						256				
Clusters		73						72						74				

a Average, Extent and Concentration refer to the Average, the Extent of deprivation and Concentration of deprivation IMD aggregate indicators by local authority. 95% CI in parentheses. *σ_u_* is the between person standard error. *σ_e_* is the model-wide error. Regression results from a model controlling for year dummies, age categories and living in a couple. Estimates from a panel fixed effects OLS regression. Standard errors from 1,000 bootstrap replications with cluster standard errors at the individual level and stratified by geographic area.

**Table 6:** Estimates of psychological/mental wellbeing (GHQ-12 score) by pensioner group after the introduction of the Minimum Income Guarantee. By 2019 IMD quintiles. Women.

		Average^a^						Extent						Concentration				
		Coef.		p-value		95% CI		Coef.		p-value		95% CI		Coef.		p-value		95% CI
**Least deprived**																		
MIG		2.58		0.526		(-5.64, 9.56)		4.22		0.115		(-1.36, 9.86)		4.24		0.091		(-1, 8.99)
MIG x Post		−0.26		0.908		(-5.1, 3.35)		0.60		0.604		(-1.9, 2.76)		0.45		0.566		(-0.92, 2.1)
*σ_u_*		4.45						4.87						5.15				
*σ_e_*		2.6						2.64						2.48				
Total		173						180						192				
Clusters		47						49						52				
**2nd quintile**																		
MIG		0.45		0.82		(-4.38, 3.8)		−2.14		0.174		(-5.22, 1.07)		−1.99		0.322		(-6.58, 1.78)
MIG x Post		−0.26		0.827		(-2.53, 2.13)		−1.59		0.171		(-3.79, 0.81)		−2.25		0.096		(-4.69, 0.44)
*σ_u_*		4.35						5.55						5.44				
*σ_e_*		2.78						2.76						3.57				
Total		196						197						202				
Clusters		57						56						57				
**3rd quintile**																		
MIG		1.51		0.274		(-1.63, 3.87)		1.87		0.223		(-1.3, 4.63)		1.68		0.316		(-1.5, 4.95)
MIG x Post		−0.02		0.987		(-1.92, 2.06)		−0.13		0.922		(-2.74, 2.62)		1.08		0.497		(-2.03, 4.25)
*σ_u_*		4.67						4.7						5.95				
*σ_e_*		3.81						3.62						3.17				
Total		349						301						200				
Clusters		95						82						59				
**4th quintile**																		
MIG		1.44		0.339		(-3.74, 3.59)		0.3		0.844		(-3.8, 2.68)		0.64		0.693		(-2.48, 4.01)
MIG x Post		0.77		0.697		(-3.68, 4.8)		0.61		0.568		(-1.51, 2.74)		0.84		0.489		(-1.22, 3.56)
*σ_u_*		5.07						4.96						4				
*σ_e_*		3.4						3.76						3.43				
Total		137						189						239				
Clusters		40						58						63				
**Most deprived**																		
MIG		−1.54		0.207		(-4.27, 0.56)		−1.44		0.255		(-3.98, 0.98)		−1.72		0.157		(-4.29, 0.36)
MIG x Post		0.22		0.832		(-1.67, 2.48)		0.38		0.717		(-1.72, 2.52)		0.67		0.485		(-1.03, 2.47)
*σ_u_*		4.73						4.52						4.98				
*σ_e_*		3.06						3.03						3.17				
Total		481						469						503				
Clusters		136						128						142				

a Average, Extent and Concentration refer to the Average, the Extent of deprivation and Concentration of deprivation IMD aggregate indicators by local authority. 95% CI in parentheses. *σ_u_* is the between person standard error. *σ_e_* is the model-wide error. Regression results from a model controlling for year dummies, age categories and living in a couple. Estimates from a panel fixed effects OLS regression. Standard errors from 1,000 bootstrap replications with cluster standard errors at the individual level and stratified by geographic area.

## Data Availability

The authors do not have permission to share data.
